# A case of recalcitrant silicone granuloma treated with adalimumab: A case report

**DOI:** 10.1177/2050313X221093444

**Published:** 2022-05-11

**Authors:** Orli M Silverberg, Benoit M Cyrenne, David Croitoru, Matthew K Sandre, Kucy Pon

**Affiliations:** 1 Temerty Faculty of Medicine, University of Toronto, Toronto, ON, Canada; 2 Division of Dermatology, Department of Medicine, University of Toronto, Toronto, ON, Canada; 3 Division of Dermatology, Women’s College Hospital, Toronto, ON, Canada; 4 Division of Dermatology, Sunnybrook Health Sciences Centre, Toronto, ON, Canada

**Keywords:** Silicone granuloma, recalcitrant silicone granuloma, adalimumab, tumour necrosis factor-α inhibitor, granulomatous inflammation

## Abstract

Liquid silicone is a relatively inexpensive injectable used for soft tissue augmentation. Injection of silicone is associated with a risk of delayed granuloma formation associated with elevated levels of tumour necrosis factor-α. We report a case of recalcitrant silicone granulomas following facial injections of silicone successfully treated with tumour necrosis factor-α blockade. Our case, as well as previous reports, demonstrates the effectiveness of this therapy for the treatment of foreign body granulomas from due to silicone.

## Introduction

Subcutaneous liquid silicone injections are a relatively inexpensive, minimally invasive approach to cosmetic alteration of facial appearance used by licenced and non-licenced practitioners.^1^ Complications include localized bruising, erythema, edema, allergic response, cellulitis, abscesses, necrosis, scarring, contractures, and granulomas.^2^ Systemic adverse events range from acute pneumonitis and hepatic granulomas to intravascular embolization leading to sudden death.^3,4^

Formation of silicone granulomas was first described in 1964 by Winer et al.^5^ as an immunologic response. They may present locally at the site of injection, but can migrate elsewhere, and usually form within 12 months.^6^ Clinically, they appear as tender nodules with induration, ulceration, and surrounding lymphadenopathy.^7^ Treatment can be challenging and without a clear consensus. Options include local and systemic steroids, tetracycline antibiotics, hydroxychloroquine, 5-fluorouracil, and isotretinoin.^8^ Surgical management is difficult and not recommended due to the tendency of silicone to migrate and cause scarring which may be more distressing than original presenting concern of granulomatous inflammation.^9^ The interferon-γ modulator, imiquimod, has been utilized, as well as tumour necrosis factor-α (TNF-α) inhibitors etanercept and adalimumab.^2,9,10^

## Case report

**Figure 1.**
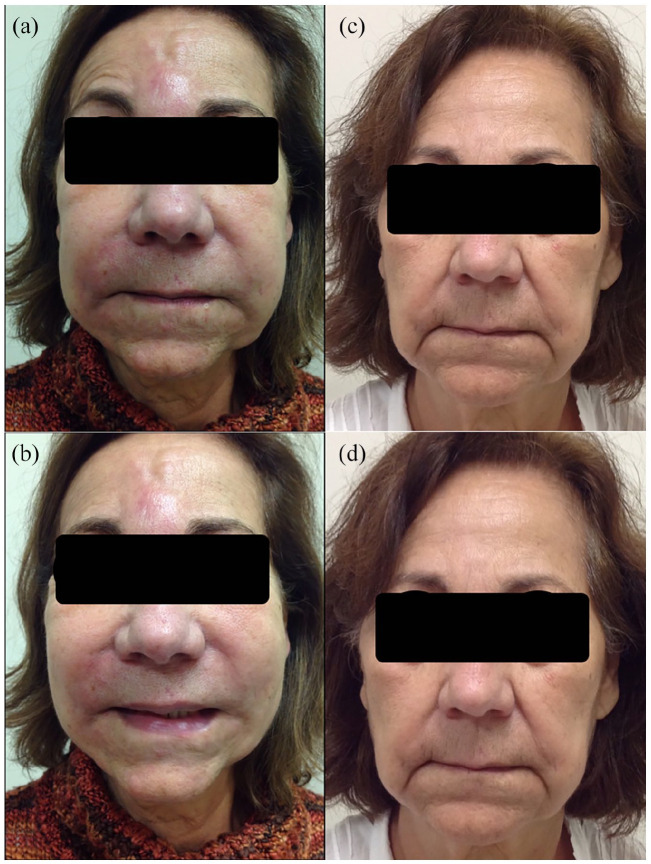
Before and after treatment of recalcitrant silicone granuloma with adalimumab. Patient on initial presentation of erythematous, tender plaques to cheeks and forehead (a), (b). Subsequent images of patient following treatment with adalimumab (c), (d).

A 78-year-old woman with a past medical history of depression and breast cancer presented with erythematous, tender plaques on her cheeks, and forehead several weeks after a dental crown repair (Figure 1(a) and (b)). Eighteen years prior, she had received silicone injections in her cheeks and glabella. Magnetic resonance imaging demonstrated findings consistent with plastic and inflammation in the areas which had been injected. She was successfully treated with a 10-day course of oral prednisone; however, she developed a flare during the taper. A skin biopsy demonstrated a granulomatous inflammation consistent with a foreign body reaction, and chest X-ray was non-contributory. She was treated with intralesional and intramuscular triamcinolone acetonide and transitioned to oral doxycycline 100 mg twice daily for 3 months, with minimal response.

She was subsequently started on oral cyclosporine, and then mycophenolate mofetil (MMF), but was unable to tolerate either medication. Repeated attempts to wean her from cyclosporine and MMF resulted in four flares requiring extended use of prednisone, cyclosporine, and MMF.

Four years after her initial presentation, the patient was started on adalimumab 40 mg subcutaneously every 2 weeks. She was weaned from prednisone, cyclosporine, and MMF and achieved a complete response. After 8 months of biologics, she developed a new exacerbation with increased facial swelling and was treated with a 2-week course of 15 mg PO daily prednisone followed by a 6-week taper. At her latest follow-up 14 months after starting adalimumab, she has not experienced any further recurrences of her symptoms (Figure 1(c) and (d)). The patient will be continued on adalimumab indefinitely.

## Discussion

The etiology of silicone granulomas is not well-understood and the variation in silicone formulations and methods of injection lead to highly heterogeneous reactions. However, it is hypothesized that T-cell activation may be initiated by the silicone itself or adulterants in the injection diluent. These granulomatous reactions are associated with elevations in the pro-inflammatory cytokine, TNF-α.^9^ Pasternack et al.^9^ described treatment of two patients with etanercept twice weekly. Similarly, Desai et al.^2^ reported a patient treated with etanercept twice weekly resulting in significant improvement in symptoms within 1 month. Our case adds to the growing evidence that TNF-α inhibition may be an important and effective modality of treatment for the otherwise recalcitrant silicone granulomas.^2,8–10^ As in the case of our patient, TNF-α inhibitors may be safer and more effective therapies than either cyclosporine or MMF. Thus, consideration should be given to initiating a TNF-α inhibitor for recalcitrant silicone granulomas before other immunomodulatory therapies.
